# Preparative Separation of Main Ustilaginoidins from Rice False Smut Balls by High-Speed Counter-Current Chromatography

**DOI:** 10.3390/toxins8010020

**Published:** 2016-01-12

**Authors:** Weibo Sun, Xuejiao Dong, Dan Xu, Jiajia Meng, Xiaoxiang Fu, Xiaohan Wang, Daowan Lai, Ligang Zhou, Yang Liu

**Affiliations:** 1 Key Laboratory of Plant Pathology, Ministry of Agriculture/Department of Plant Pathology, College of Plant Protection, China Agricultural University, Beijing 100193, China; sunweibo.1001@163.com (W.S.); 18874783364@163.com (X.D.); cauxudan@163.com (D.X.); mengjiajiax@163.com (J.M.); xiaoxiaofu@cau.edu.cn (X.F.); wangxiaohan99@126.com (X.W.); dwlai@cau.edu.cn (D.L.); 2 Institute of Food Science and Technology, Chinese Academy of Agricultural Sciences/Key Laboratory of Agro-products Processing, Ministry of Agriculture, Beijing 100193, China

**Keywords:** ustilaginoidins, mycotoxin, preparative separation, rice false smut balls, *Villosiclava virens*, *Ustilaginoidea virens*, high-speed counter-current chromatography

## Abstract

Ustilaginoidins are bis-naphtho-γ-pyrone mycotoxins isolated from the rice false smut balls (FSBs) infected by the pathogen *Villosiclava virens* in rice spikelets on panicles. In order to obtain large amounts of pure ustilaginoidins to further evaluate their biological activities and functions, phytotoxicity on rice, security to human and animals as well as to accelerate their applications as pharmaceuticals, preparative high-speed counter-current chromatography (HSCCC) was successfully applied to the isolation and purification of seven bis-naphtho-γ-pyrone mycotoxins, namely ustilaginoidins A (**1**), G (**2**), B (**3**), H (**4**), I (**5**), C (**6**), and J (**7**) from the ethyl acetate crude extract of rice FSBs. Both **1** and **2** were prepared by HSCCC from the low-polarity fraction of the crude extract using the two-phase solvent system composed of *n*-hexane-ethyl acetate-methanol-water at the volume ratio of 6.5:3.5:5.0:5.0. Similarly, **3**, **4** and **5** were prepared from the medium-polarity fraction using the system at the volume ratio of 4.0:5.0:5.0:6.0, and **6** and **7** were prepared from the higher-polarity fraction using the system at volume ratio of 3.0:5.0:4.0:6.7. A total of 6.2 mg of **1**, 5.1 mg of **2**, 3.9 mg of **3**, 1.2 mg of **4**, 5.7 mg of **5**, 3.5 mg of **6**, and 6.1 mg of **7** with purities of 88%, 82%, 91%, 80%, 92%, 81% and 83%, respectively, were yielded from total 62 mg fraction samples in three independent HSCCC runs. The structures of the purified ustilaginoidins were characterized by means of physicochemical and spectrometric analysis.

## 1. Introduction

The rice false smut caused by the ascomycetous pathogen *Villosiclava virens* (Nakata) Tanaka and Tanaka (anamorph: *Ustilaginoidea virens* Takahashi) is an emerging, increasingly significant and worldwide fungal disease in many rice (*Oryza sativa* L.) cultivation areas over the past few years [1,2]. This disease was once considered a minor disease with low frequency of occurrence in rice-growing areas. However, as a result of extensive planting of high-yielding cultivars and hybrids, overuse of chemical fertilizers and an apparent change in global and regional climates, rice false smut has recently become one of the most devastating grain diseases in the majority of rice-growing areas of the world [3,4,5,6]. In particular, this disease has been estimated to occur in one-third of the rice cultivation areas in China [2].

A key characteristic of rice false smut is the formation of ball-like colonies, which are called rice false smut balls (FSBs), in the spikelets during the late phase of pathogen infection [7]. With regard to the chemical constituents of the FSBs, there are two types of mycotoxins (*i.e.*, ustiloxins and ustilaginoidins) that have been isolated from the balls [8,9,10,11,12]. Ustilaginoidins are red or yellow mycotoxins isolated from the ethyl acetate extract of the rice FSBs. They possess the same skeleton structure, which consists of two naphtho-γ-pyrones connected by the 9-9′ single bond, and their absolute configurations have been determined to be *R* according to the Cotton effects shown from circular dichroism (CD) [13].

There are some reports available in the literature regarding to the biological activities of ustilaginoidins [14,15,16,17]. Ustilaginoidin A showed inhibitory activity on mouse embryo limb bud (LB) and midbrain (MB) cells with IC_50_ values of 1.30 and 0.64 µg/mL, respectively [14]. Ustilaginoidin A also inhibited ATP synthesis in mitochondria by uncoupling oxidative phosphorylation and depressing state-3 respiration of mitochondria [15]. Ustilaginoidins A, B, G, H, I and J were found to have cytotoxic activity on human epidermoid carcinoma (KB) cells with IC_50_ values of 0.3, 22.5, 1.0, 54, 13.5 and 60 µg/mL, respectively [16]. Ustilaginoidin G showed inhibitory effect against six pathogenic bacteria with IC_50_ values ranged from 3.25 to 10.64 µg/mL [17]. The potential applications of ustilaginoidins as antitumor agents and antimicrobials as well as their promising bioactivities have led to considerable interest in the pharmaceutical community [12]. However, the biological functions of ustilaginoidins during the occurrence and spread of rice false smut along with their potential risks to grain production and food safety remain unclarified. Thus, it is necessary to obtain large amounts of pure ustilaginoidins to further evaluate their biological activities and functions, phytotoxicity on rice, security to human and animals as well as to accelerate their applications as pharmaceuticals.

**Figure 1:**
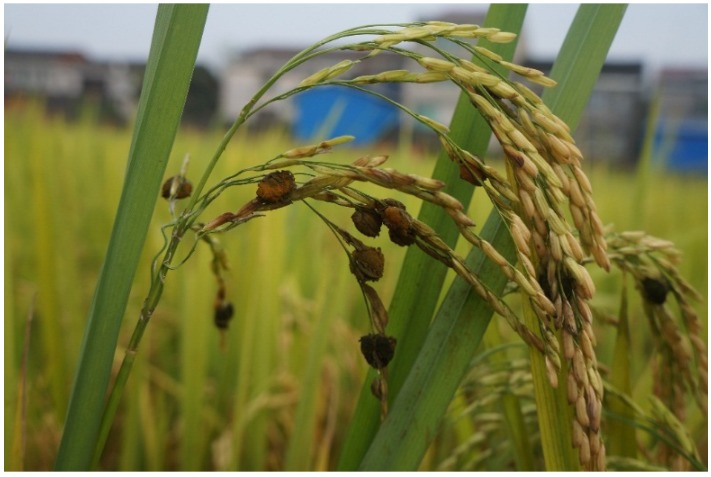
Rice false smut balls (FSBs) formed in the late growth stage of rice infected by the pathogen *Villosiclava virens*.

Rice FSBs can be obtained conveniently from the rice field in the late growth stage of rice infected by the pathogen *Villosiclava virens* (Figure 1). They have been considered as the main source of ustilaginoidins. The structures of ustilaginoidins are similar to each other, especially with six hydroxyl groups binding on their naphthalene rings at the positions of *C*-5, 6, 8, 5′, 6′ and 8′. Though we can get the ustilaginoidins from rice FSBs by classical methods, those methods are time- and solvent-consuming, and require multiple chromatographic steps on silica gel, Sephadex LH-20, *etc.* [17]. Thus, it is necessary to search for an effective and rapid method for the purification of ustilaginoidins from the rice FSBs.

High-speed counter-current chromatography (HSCCC) is a unique liquid–liquid partition chromatography technique that requires no solid support matrix. It overcomes the irreversible adsorptive loss of samples onto the solid support matrix and also has the unique features of high recovery, high efficiency and ease of scaling [18]. This method has been successfully applied to separate and purify a number of natural products from a variety of plant and microbial materials [19,20,21,22,23,24].

**Figure 2:**
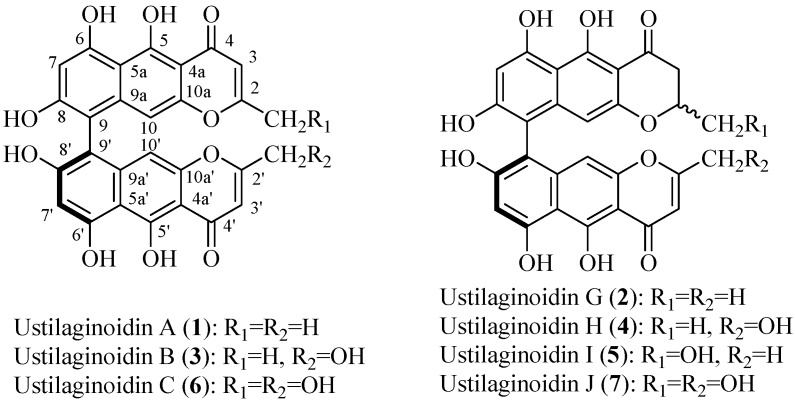
Chemical structures of the seven main ustilaginoidins (**1**–**7**) isolated from the rice FSBs by using high-speed counter-current chromatography (HSCCC).

To the best of our knowledge, there was no previous report on the preparative separation of ustilaginoidins from natural materials. The aim of this study was to establish a method for more efficient separation and purification of ustilaginoidins from the rice FSBs by HSCCC. Seven HSCCC-separated ustilaginoidins (Figure 2) were further purified by semi-preparative HPLC, and structurally characterized by means of physicochemical and spectrometric analysis.

## 2. Results and Discussion

### 2.1. HPLC Analysis of the Crude Extract

**Figure 3:**
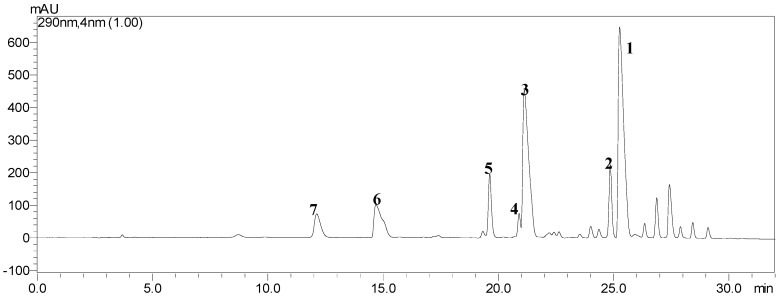
HPLC chromatogram of the ethyl acetate crude extract from the rice FSBs. The retention times of seven peaks corresponding to ustilaginoidins A (**1**), G (**2**), B (**3**), H (**4**), I (**5**), C (**6**) and J (**7**) were 25.27, 24.85, 21.12, 20.90, 19.63, 14.70 and 12.10 min, respectively.

The ethyl acetate (EtOAc) crude extract obtained from the rice FSBs was firstly analyzed by a high performance liquid chromatography (HPLC) system eluted with a linear gradient of methanol from 50% to 100% (*v*/*v*) and water (containing 0.01% oxalic acid) from 50% to 0 (*v*/*v*) over 32 min at a flow rate of 1.0 mL/min. The temperature was maintained at 30 °C, UV detection at 290 nm, and the sample injection volume as 10 μL. The LC-solution multi-PDA workstation was employed to acquire and process chromatographic data. Under the above conditions, a satisfactory separation of the crude extract was obtained, and the HPLC chromatogram of the crude extract is shown in Figure 3. Seven peaks corresponding to ustilaginoidins A (**1**), G (**2**), B (**3**), H (**4**), I (**5**), C (**6**) and J (**7**), which were structurally characterized by means of physicochemical and spectrometric analysis (see Section 2.4).

### 2.2. Selection of the Suitable Two-Phase Solvent System

Appropriate solvent system plays an essential role in HSCCC separation. It can provide the ideal partition coefficient (*K* value) for the target compounds, which is a crucial parameter for the separation, and the satisfied *K* value is usually considered in the range of 0.5–2.0 [25]. If the *K* value is too small, the compounds will be eluted closely to the solvent front with poor resolution, while the higher *K* value tends to lead a good resolution but it will cause broad peaks and a longer elution time. In this study, ustilaginoidins belong to medium polar compounds, which can be dissolved in ethyl acetate, acetone, and slightly in methanol, but toughly dissolved in alkane and water. We tried to directly purify ustilaginoidins from the EtOAc crude extract by HSCCC but failed. It was found that if the EtOAc crude extract was first grouped into three fractions (Frs. A, B and C) according to their polarities by silica gel and Sephadex LH-20 column chromatography, the efficient separations could be succeeded when these fraction samples were independently separated by HSCCC chromatography.

The preliminary studies were carried out to determine the *K* values of the target compounds in the most widely used two-phase solvent systems composed of *n*-hexane-ethyl acetate-methanol-water at various volume ratios of 3.0:5.0:3.0:5.0, 3.0:5.0:4.0:6.7, 4.0:5.0:4.0:5.0, 4.0:5.0:5.0:6.0, 6.5:3.5:5.0:5.0, and 7.0:3.0:5.0:5.0, coded as S1–S6, respectively, by HPLC analysis. The results are summarized in Table 1.

**Table 1:** The partition coefficients (*K* values) of seven ustilaginoidins in two-phase solvent systems of *n*-hexane-ethyl acetate-methanol-water by HPLC analysis.

System No.	Ratio (*v*/*v*)	*K* Value						
		1	2	3	4	5	6	7
S1	3.0:5.0:3.0:5.0	+	+	2.77	2.81	2.25	1.33	1.13
S2	3.0:5.0:4.0:6.7	+	+	2.72	2.90	2.46	1.25	0.92
S3	4.0:5.0:4.0:5.0	5.17	5.49	2.35	2.65	2.31	1.29	1.16
S4	4.0:5.0:5.0:6.0	2.23	2.45	1.07	1.28	0.72	0.39	0.34
S5	6.5:3.5:5.0:5.0	0.89	1.26	0.12	0.13	0.08	–	–
S6	7.0:3.0:5.0:5.0	0.58	0.61	–	–	–	–	–

Note: “Ratio” is expressed as the volume ratio of *n*-hexane-ethyl acetate-methanol-water. “+” or “–” means the *K* value of each compound in the solvent is either too big or too small that it is not suitable for the isolation in HSCCC. Compounds **1**–**7** correspond to ustilaginoidins A, G, B, H, I, C and J, respectively.

The results indicated that when the two-phase solvent system S2 at a volume ratio of 3.0:5.0:4.0:6.7 was used, the higher-polarity Fr.C contained **6** and **7** with the *K* values of 1.25 and 0.92 could be separated at an appropriate time. However, using the solvent system S2, both low-polarity fraction Fr.A (containing **1** and **2**) and medium-polarity fraction Fr.B (containing **3**–**5**) would be retained in the column for a long time as a result of their big *K* values. On the other hand, when the two-phase solvent system S6 at a volume ratio of 7.0:3.0:5.0:5.0 was used, Fr.A could be separated in a short elution time, but other fractions were most tend to elute closely with the solvent front without any resolution as their *K* values were too small. Finally, we selected the solvent system S2 composed of *n*-hexane-ethyl acetate-methanol-water at a volume ratio of 3.0:5.0:4.0:6.7 as the appropriate system for further separation of compounds **6** and **7** from Fr.C, the system S4 at a volume ratio of 4.0:5.0:5.0:6.0 for further separation of compounds **3**, **4** and **5** from Fr.B, and the system S5 at a volume ratio of 6.5:3.5:5.0:5.0 for further separation of compounds **1** and **2** from Fr.A.

### 2.3. HSCCC Separation of the Samples

**Figure 4:**
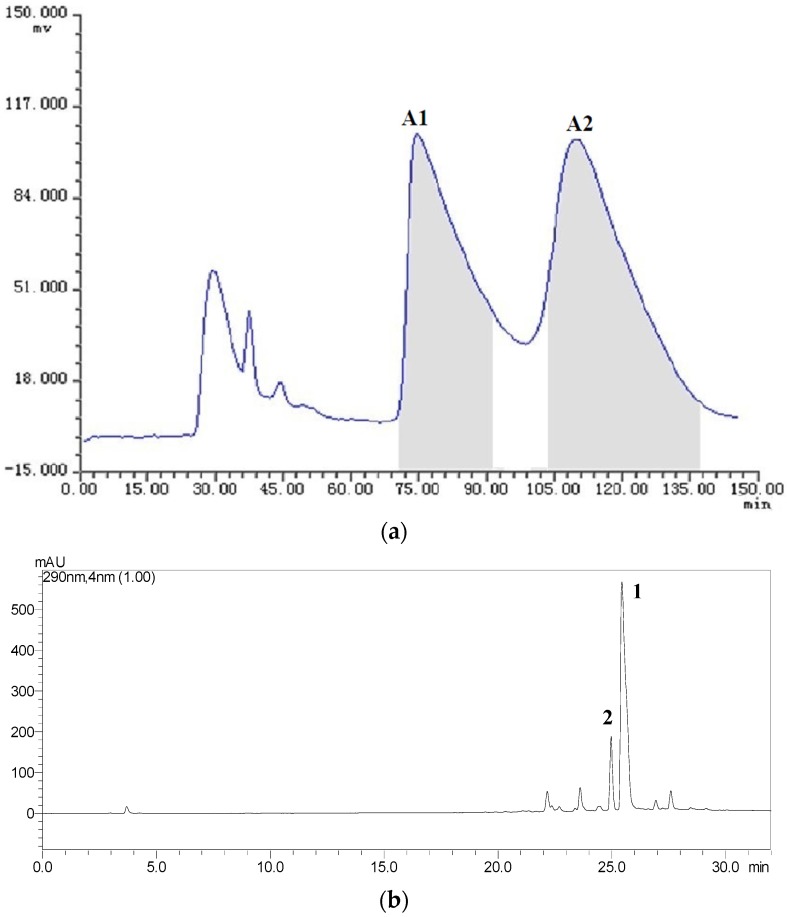

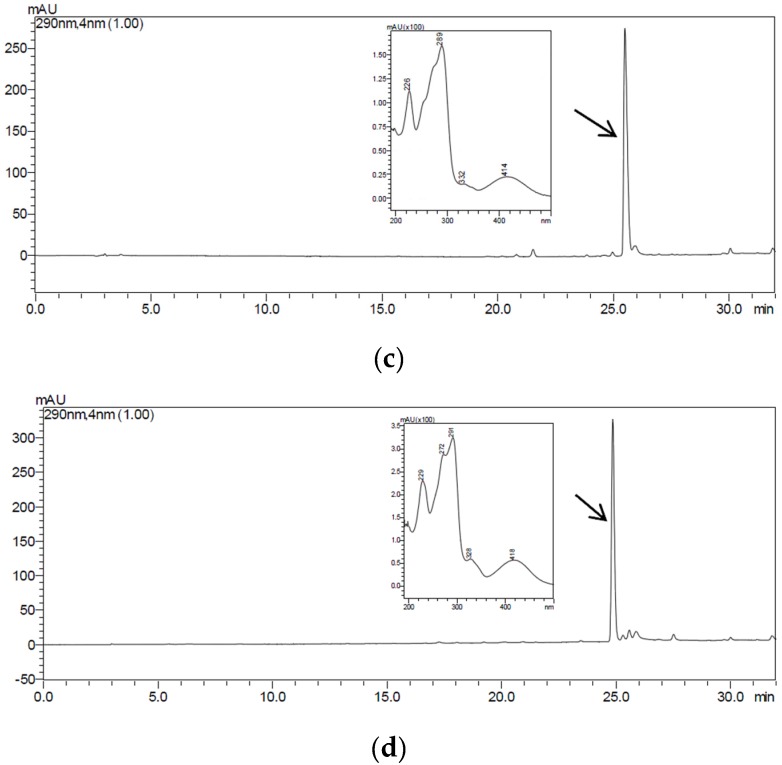
HSCCC chromatogram of Fr.A along with the HPLC analysis and UV spectra of HSCCC peak fractions: (**a**) HSCCC chromatogram of Fr.A with *n*-hexane-ethyl acetate-methanol-water (6.5:3.5:5.0:5.0, *v*/*v*) solvent system; (**b**) HPLC chromatogram of Fr.A before HSCCC; (**c**) ustilaginoidin A (**1**) from HSCCC peak fraction A1; and (**d**) ustilaginoidin G (**2**) from HSCCC peak fraction A2. The HPLC retention time of A1 and A2 was 25.27 and 24.85 min, respectively.

**Figure 5:**
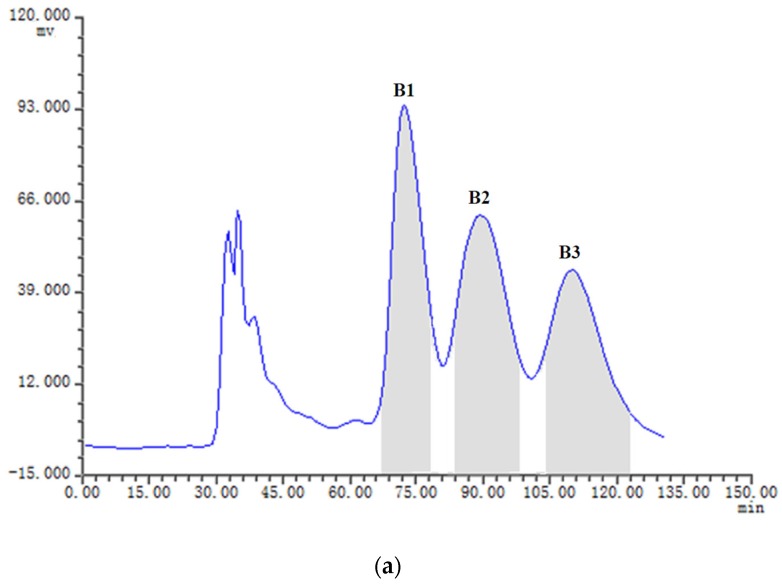

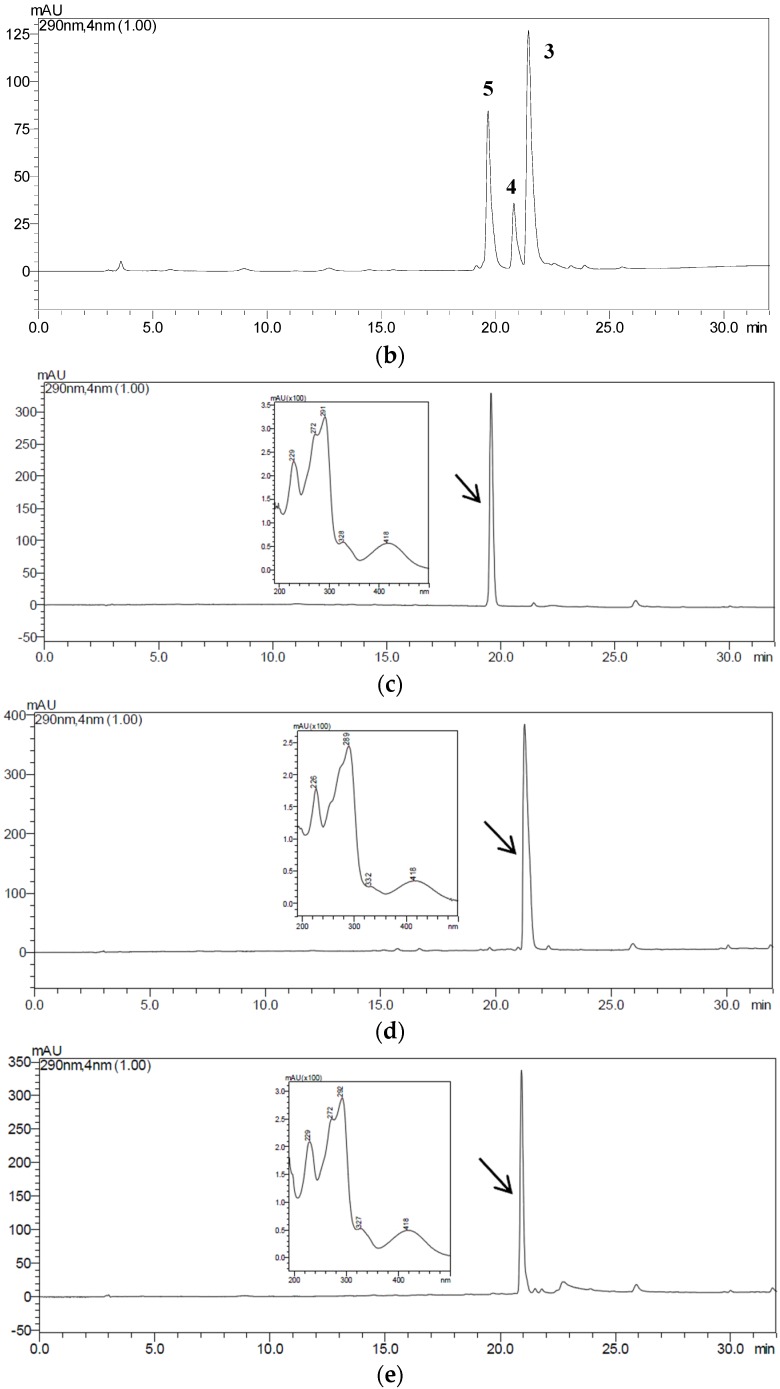
HSCCC chromatogram of Fr.B along with the HPLC analysis and UV spectra of HSCCC peak fractions: (**a**) HSCCC chromatogram of Fr.B with *n*-hexane-ethyl acetate-methanol-water (4.0:5.0:5.0:6.0, *v*/*v*) solvent system; (**b**) HPLC chromatogram of Fr.B before HSCCC; (**c**) ustilaginoidin I (**5**) from HSCCC peak fraction B1; (**d**) ustilaginoidin B (**3**) from HSCCC peak fraction B2; and (**e**) ustilaginoidin H (**4**) from HSCCC peak fraction B3. The HPLC retention time of B1, B2 and B3 was 19.63, 21.12 and 20.90 min, respectively.

**Figure 6:**
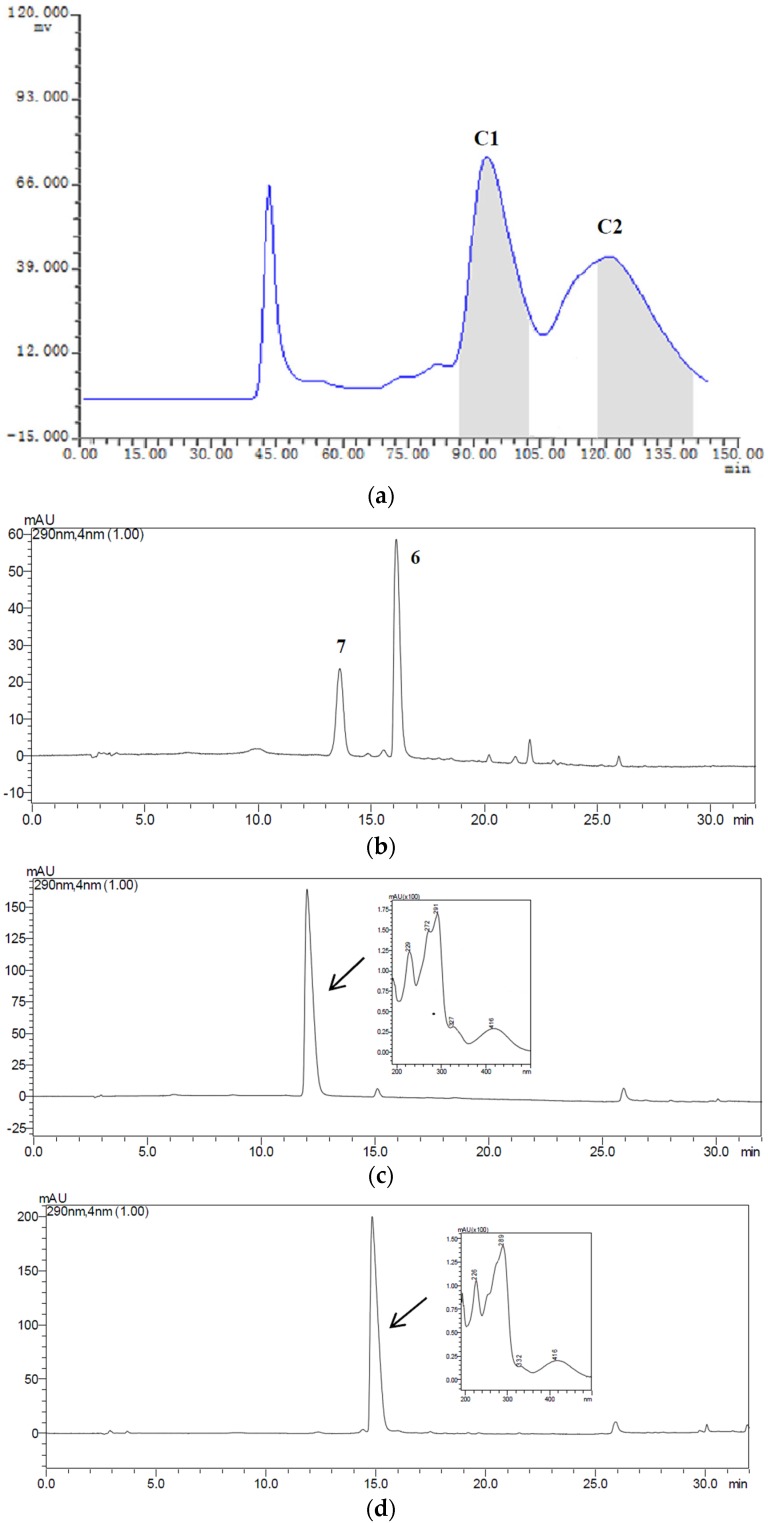
HSCCC chromatogram of Fr.C along with the HPLC analysis and UV spectra of HSCCC peak fractions: (**a**) HSCCC chromatogram of Fr.C with *n*-hexane-ethyl acetate-methanol-water (3.0:5.0:4.0:6.7, *v*/*v*) solvent system; (**b**) HPLC chromatogram of Fr.C before HSCCC; (**c**) ustilaginoidin J (**7**) from HSCCC peak fraction C1; and (**d**) ustilaginoidin C (**6**) from HSCCC peak fraction C2. The HPLC retention time of C1 and C2 was 12.10 and 14.70 min, respectively.

The samples Fr.A (low-polarity fraction), Fr.B (medium-polarity fraction) and Fr.C (higer-polarity fraction) containing two or three target compounds were separated by HSCCC with three different solvent systems according to the *K* values mentioned in Table 1. The HSCCC separation was achieved in the head to tail elution mode and the upper phase of the two-phase solvent system was employed as the stationary phase. The separation was considered to be efficient as the retention ratios (*S*_F_) of the stationary phases for the systems S2, S4 and S5 were 76.31%, 74.02% and 72.79%, respectively [18]. About 19 mg of Fr.A, 21 mg of Fr.B, and 22 mg of Fr.C were independently dissolved in 12 mL of mobile phase, and were then injected into the equilibrium system and separated by HSCCC with their correspondent two-phase solvent systems S5, S4 and S2. The effluent from the outlet of the column was monitored with the UV detector at 280 nm, and the fractions were manually collected according to the chromatograms. The fractions were analyzed by HPLC, and those of same pure compound were combined and evaporated under reduced pressure. The HSCCC chromatograms for the isolation of Fr.A, Fr.B and Fr.C are shown in Figure 4a, Figure 5a and Figure 6a, respectively. We found that the two peaks A1 and A2 in Fr.A were finely separated when using S5 (*n*-hexane-ethyl acetate-methanol-water as 6.5:3.5:5.0:5.0, *v*/*v*) as the solvent system in HSCCC procedure. The peak fractions A1 and A2 collected during 70–91 min and 106–140 min were evaporated and determined to be ustilaginoidins A (**1**) and G (**2**) with purity of 88% and 82%, respectively, analyzed by HPLC (Figure 4c,d). The similar separations were also obtained when using the systems S4 (*n*-hexane-ethyl acetate-methanol-water as 4.0:5.0:5.0:6.0, *v*/*v*) for Fr.B, and S2 (*n*-hexane-ethyl acetate-methanol-water as 3.0:5.0:4.0:6.7, *v*/*v*) for Fr.C. The peak fractions B1, B2 and B3 collected during 67–78 min, 85–98 min and 104–124 min in Fr.B (Figure 5a) were ustilaginoidins I (**5**), B (**3**) and H (**4**) with purity of 92%, 91% and 80%, respectively, analyzed by HPLC (Figure 5c–e). The peak fractions C1 and C2 collected during 88–101 min and 118–140 min in Fr.C (Figure 6a) were peak ustilaginoidins J (**7**) and C (**6**) with purity of 83% and 81%, respectively, analyzed by HPLC (Figure 6c,d). The purities and quantities of the compounds before and after HSCCC are shown in Table 2. The running time for each HSCCC separation procedure was less than 150 min.

**Table 2:** Purities and quantities of the compounds before and after HSCCC.

Fraction Sample and Quantity	HSCCC Peak Fraction and Corresponding Compound	Purity before HSCCC (%)	Purity after HSCCC (%)	Quantity of HSCCC Peak Fraction (mg)
Fr.A, 19 mg	A1 (1)	35	88	6.2
	A2 (2)	29	82	5.1
Fr.B, 21 mg	B1 (5)	28	92	5.7
	B2 (3)	31	91	3.9
	B3 (4)	12	80	1.2
Fr.C, 22 mg	C1 (7)	31	83	6.1
	C2 (6)	26	81	3.5

Note: Compounds **1**–**7** correspond to ustilaginoidins A, G, B, H, I, C and J, respectively.

We could conclude that the employment of HSCCC is good way to isolate ustilaginoidins with high purities, especially for the compounds that could not be completely isolated using the traditional methods. In addition, HSCCC has other advantages such as lower solvent consumption, avoiding the loss of samples and reducing runtime. Considering that HSCCC is a liquid–liquid partition chromatography technique, the outflow order of the compounds in HSCCC is different from the solid support based column chromatography in HPLC. Although the amounts of samples dissolved in the mobile phase are not very large, we can repeat the HSCCC procedures to obtain enough ustilaginoidins.

### 2.4. Purification and Structural Identification of the Compounds

In order to obtain sufficient samples with the high enough purity to meet the needs of structural identification by MS and NMR analysis, we repeated the HSCCC procedures and accumulated the seven compounds for further semi-preparative HPLC purification. They were repeatedly performed on a reversed-phase C_18_ semi-preparative column with an isocratic elution of methanol-water (volume ratio of 50:50 for peaks C1 and C2 separation in Fr.C; volume ratio of 65:35 for peaks B1, B2 and B3 separation in Fr.B; and volume ratio of 75:25 for peaks A1 and A2 separation in Fr.A) at a flow rate of 3.0 mL/min.

All the purified compounds were isolated as red or yellow amorphous powder. The subsequent structural identifications of these compounds were performed by HR-ESI-MS, ^1^H-NMR and ^13^C-NMR spectra. The molecular formula C_28_H_18_O_10_ of compound **1** from the HSCCC peak fraction A1 was assigned by HR-ESI-MS at *m*/*z* 513.0831 [M − H]^−^ for (calcd for C_28_H_17_O_10_, 513.0827). The molecular formula C_28_H_20_O_10_ of compound **2** from the HSCCC peak fraction A2 was assigned by HR-ESI-MS at *m*/*z* 515.0987 [M − H]^−^ for (calcd for C_28_H_19_O_10_, 515.0984). The molecular formula C_28_H_18_O_11_ of compound **3** from the HSCCC peak fraction B2 was assigned by HR-ESI-MS at *m*/*z* 531.0903 [M + H]^+^ (calcd for C_28_H_19_O_11_, 531.0922). The molecular formula C_28_H_20_O_11_ of compound **4** from the HSCCC peak fraction B3 was assigned by HR-ESI-MS at *m*/*z* 533.1078 [M + H]^+^ (calcd for C_28_H_21_O_11_, 533.1078). The molecular formula C_28_H_20_O_11_ of compound **5** from the HSCCC peak fraction B1 was assigned by HR-ESI-MS at *m*/*z* 533.1070 [M + H]^+^ (calcd for C_28_H_21_O_11_, 533.1078). The molecular formula C_28_H_18_O_12_ of compound **6** from the HSCCC peak fraction C2 was assigned by HR-ESI-MS at *m*/*z* 547.0856 [M + H]^+^ (calcd for C_28_H_19_O_12_, 547.0871). The molecular formula C_28_H_20_O_12_ of compound **7** from the HSCCC peak fraction C1 was assigned by HR-ESI-MS at *m*/*z* 549.1015 [M + H]^+^ (calcd for C_28_H_19_O_12_, 549.1028).

The data of ^1^H-NMR (600 MHz) and ^13^C-NMR (150 MHz) for compounds **1**–**7** are shown in Tables S1 and S2. The measured spectra data were compared with the previous reports [11,17]. The peaks A1 and A2 in Fr.A were identified as ustilaginoidins A (**1**) and G (**2**), respectively. The peaks B1, B2 and B3 in Fr.B were identified as ustilaginoidins I (**5**), B (**3**), H (**4**), respectively. The peaks C1 and C2 in Fr.C were ustilaginoidins J (**7**) and C (**6**), respectively.

It is notable that the seven compounds all share the same skeleton structure that consists of two naphtho-γ-pyrones connected by the 9-9′ single bond. They have an *R* configuration of the 9,9′-axis, and only differ in the γ-pyrone rings which are substituted with either methyl groups or hydroxymethyl groups at *C*-2 or *C*-2′. However, ustilaginoidins A (**1**) and G (**2**) with the methyl groups at *C*-2 and *C*-2′ have been isolated from the fermentation cultures of the pathogen *U. virens* in our previous study [17]. The ustilaginoidins with hydroxymethyl groups at *C*-2 or *C*-2′ have not been isolated from the fermentation cultures of the pathogen. It seems that ustilaginoidins B (**3**), H (**4**), I (**5**), C (**6**), and J (**7**) with hydroxymethyl groups at *C*-2 or *C*-2′ were only found in the rice FSBs formed in the natural rice field. These ustilaginoidins with a high degree of oxidation in the rice FSBs may have their physiological and ecological functions which need to be studied in detail.

## 3. Experimental Section

### 3.1. General Experimental Procedures

The preparative high-speed counter-current chromatography (HSCCC) employed in this study was a TBE-300B instrument (Tauto Biotech Co. Ltd., Shanghai, China). The apparatus with the maximum rotational speed of 1000 rpm was equipped with three polytetrafluoroethylene preparative coils (ID 2.6 mm, total volume is 300 mL), and a 20 mL manual sample loop. The HSCCC system consists of a TBP-5002 pump, a TBD-2000 UV detector, a WH500-USB chromatography workstation (Wuhao, Shanghai, China), and a HX-1050 constant temperature regulator (Beijing Boyikang Laboratory Instruments, Beijing, China). The separation was carried out at 25 °C using a two-phase solvent system, at flow rate of 3.2 mL/min, revolution speed of 800 rpm, and detection wavelength at 280 nm.

The analytical Prominence LC-20A high-performance liquid chromatography (HPLC) system (Shimadzu, Kyoto, Japan) consisted of two LC-20AT solvent delivery units, an SIL-20A autosampler (Shimadzu, Kyoto, Japan), an SPD-M20A photodiode array detector (Shimadzu, Kyoto, Japan), and CBM-20 Alite system controller (Shimadzu, Kyoto, Japan). Chromatographic separations were performed at 30 °C using Luna reversed-phase C_18_ column (250 mm × 4.6 mm, 5 μm, Phenomenex, Torrance, CA, USA). The mobile phase consisted of methanol (A), and water containing 0.01% oxalic acid (B), with gradient elution of 50% A hold at 0–5 min, 50% A rose to 100% A at 5–25 min, and 100% A hold at 25–32 min. The flow rate was 1.0 mL/min, and the effluent was monitored at 190–500 nm. The LC solution multi-PDA workstation was employed to acquire and process chromatographic data. Total period of analysis was 32 min.

The semi-preparative HPLC system consisted of K-501 pump, K-2501 UV detector (Knauer, Berlin, Germany), a 2 mL sample loop, a chromatography workstation (Lumtech, Beijing, China), and a Luna reversed-phase C_18_ column (250 mm × 10 mm, 5 μm, Phenomenex, Torrance, CA, USA). The mobile phase consisted of methanol (A), and water (B), with constant elution of 50% or 65% or 75% A hold at 0–30 min for different polarities of the compounds. The flow rate was 3.0 mL/min, and the effluent was monitored at 290 nm.

HR-ESI-MS spectra were measured on Bruker Apex IV FTMS (Bruker Daltonics, Bremen, Germany). NMR spectra (^1^H-NMR and ^13^C-NMR) were recorded on a Bruker Avance DRX-NMR spectrometer (^1^H at 600 MHz and ^13^C at 150 MHz) (Bruker BioSpin, Zurich, Switzerland). The chemical shifts were expressed in ppm as δ values relative to tetramethylsilane (TMS) as an internal standard.

A KH-500E ultrasonic cleaner was purchased from Kunshan Hechuang Ultrasonic Apparatus Co. Ltd. (Kunshan, China). Silica gel (200–300 mesh) for column chromatography was purchased from the Qingdao Marine Chemical Company (Qingdao, China). Sephadex LH-20 was purchased from Pharmacia Biotech, Uppsala Sweden.

All organic solvents used for HSCCC separation were of analytical grade and purchased from Beijing Chemical Company (Beijing, China). Methanol used for semi-preparative and analytical HPLC was chromatography grade and was purchased from Xilong Chemical Company (Shantou, China). Ultrapure water was used throughout the experiment. All other chemicals and reagents were of analytical grade.

### 3.2. Rice False Smut Balls

The rice false smut balls (FSBs) were collected from Linyi (118.24°E, 35.15°N), the southwest of Shandong Province in China in October 2011. The materials were left to dry in shade at room temperature to a constant weight, and were stored in the sealed plastic bags at −20 °C until required.

### 3.3. Preparation of the Crude Extract

The dry and powdered rice FSBs (9.1 kg) were soaked in deionized water at room temperature for three times (3 × 30 L, 48 h for each time) and shaken vigorously occasionally. After filtration, the residue was soaked in ethanol at room temperature for another three times (3 × 30 L, 48 h for each time). The ethanol filtrates were combined and concentrated under vacuum to obtain a black gum-like substance which was suspended in water and extracted first with the equal volume of petroleum ether, and then with ethyl acetate (EtOAc), and last with *n*-butanol for three times. The combined EtOAc solution was concentrated to obtain a red extract (264.1 g) which was further chromatographed on a silica gel column eluted with a step gradient of CH_2_Cl_2_-EtOAc (1:0, 100:1, 10:1, 1:1, and 0:1, *v*/*v*) to yield two main fractions Fr.A (27.5 g) and Fr.M (43.5 g). One part (10.0 g) of Fr.M was further fractionated on a Sephadex LH-20 column eluted with CHCl_3_-CH_3_OH (1:1, *v*/*v*) to afford Fr.B (5.2 g) and Fr.C (3.6 g). The subsequent isolations for Fr.A (low-polarity fraction), Fr.B (medium-polarity fraction) and Fr.C (higher-polarity fraction) were performed separately on HSCCC.

### 3.4. Measurement of Partition Coefficient

The two-phase solvent systems were screened according to the partition coefficients (*K* values) of the target compounds [23]. The *K* values were determined by HPLC analysis as follows. The suitable sample was weighed and put into a 50 mL-separating funnel, and then an equal volume (20 mL) of the equilibrated upper and lower phases of the two-phase solvent system was added to the funnel, respectively. Then the funnel was sealed and shaken vigorously for 1 min to thoroughly equilibrate the compounds between the upper and lower phases. Five milliliters of either the upper or lower phase was transferred and evaporated. The residue was dissolved in 1 mL methanol and analyzed by HPLC to obtain the partition coefficient (*K* value). The *K* value was defined as the peak area of compound in the upper phase divided by the peak area of the compound in the lower phase.

### 3.5. Preparation of the Solvent Systems and Sample Solutions

The optimized two-phase solvent systems consisted of *n*-hexane-ethyl acetate-methanol-water with the volume ratio of 6.5:3.5:5.0:5.0, 4.0:5.0:5.0:6.0 and 3.0:5.0:4.0:6.7, respectively, were used for HSCCC separation. Each two-phase solvent system was thoroughly equilibrated in a separating funnel by vigorously shaking at room temperature. Then the solvent system was left overnight and the two parts were separated and degassed by sonication for 30 min shortly prior to use. The upper part was used as the stationary phase, and lower part was used as the mobile phase. The sample solution was prepared through a sonication-assisted dissolution of the samples in 12 mL solvent of the lower phase, then the cleared solution was obtained in the mini-centrifuge for the further HSCCC isolation and separation.

### 3.6. HSCCC Separation Procedure

For the operation of the HSCCC apparatus, the multilayer coil column was first completely filled with the upper phase of the two-phase solvent system as the stationary phase. Then the HSCCC apparatus was rotated at a revolution speed of 800 rpm. Meanwhile, the lower phase was pumped into the coil column at a flow rate of 3.2 mL/min as the mobile phase. After the mobile phase front was emerged and hydrodynamic equilibrium was established throughout the column, 12 mL sample solution was injected into the column. The effluent of the column was continuously monitored by a UV detector at 280 nm and each peak fraction was manually collected according to the chromatogram. The separation temperature was controlled at 25 °C during the whole experiment. After the separation, the solvents in the column were pushed out using the N_2_ airflow.

The retention ratio (*S*_F_) of stationary phase was also calculated, and the equation was *S_F_* (%) = [(322 − *V_S_*) × 100]/(305 − 20), where *S*_F_ is the retention ratio of stationary phase (upper phase), and *V_S_* is the volume of stationary phase flowing out [22].

### 3.7. HPLC Analysis and Identification of Ustilaginoidins

The crude extract sample and peak fractions were analyzed by HPLC with a Luna reversed-phase C_18_ column as mentioned above. Methanol and water (with 0.01% oxalic acid) were used as the mobile phase and the peaks were eluted with the gradient elution mode. The compounds were monitored at 190–500 nm and their UV-profiles were provided.

In order to quantitatively analyze the content of each compound, 1 mg of the purified ustilaginoidin was dissolved in 1 mL of methanol to obtain the mother solution (1 mg/mL), which was further diluted into a series of concentrations of 150, 100, 50, 31.25, 25, 12.5, 6.25 and 3.125 μg/mL with methanol, and the diluted solutions were kept at 4 °C. The linear equations of the compounds by HPLC analysis were expressed as *Y* = a*X* + b (*R*^2^), where *Y* was the peak area, *X* was the quantity (μg) of the compound injected each time (10 μL of the injected solution), and *R* was the correlation coefficient. The linear equations were *Y* = 2687719.1*X* + 20923.6 (*R*^2^ = 1.0000) for ustilaginoidin A (**1**), *Y* = 5284024.8*X* + 120964.7 (*R*^2^ = 0.9990) for ustilaginoidin G (**2**), *Y* = 10988456.1*X* + 65940.6 (*R*^2^ = 1.0000) for ustilaginoidin B (**3**), *Y* = 5034309.9*X* + 71546.1 (*R*^2^ = 0.9999) for ustilaginoidin H (**4**), *Y* = 5108967.5*X* + 89245.9 (*R*^2^ = 0.9994) for ustilaginoidin I (**5**), *Y* = 6125884.7*X* + 104176.0 (*R*^2^ = 0.9995) for ustilaginoidin C (**6**), and *Y* = 5792811.6*X* + 86644.5 (*R*^2^ = 0.9998) for ustilaginoidin J (**7**).

The identifications of the isolated compounds were performed according to the HR-ESI-MS data as well as ^1^H and ^13^C-NMR data. The measured data were compared with the reported references to confirm the structures of the compounds [11,17].

## 4. Conclusions

In this study, an efficient HSCCC method was developed for the preparative separation of seven bis-naphtho-γ-pyrones, namely ustilaginoidins A (**1**), G (**2**), B (**3**), H (**4**), I (**5**), C (**6**), and J (**7**), from the rice FSBs for the first time. The crude extract was fractionated using silica gel and Sephadex LH-20 column chromatography to obtain three fractions (Frs.A, B and C) according to their polarities, then three two-phase solvent systems composed of *n*-hexane-ethyl acetate-methanol-water at the volume ratio of 6.5:3.5:5.0:5.0, 4.0:5.0:5.0:6.0 and 3.0:5.0:4.0:6.7 were selected to isolate the compounds, respectively. The compounds could be finely separated within the 150 min procedure in each run. The obtained ustilaginoidins might be used to further evaluate their biological activities and functions, phytotoxicity on rice, security to human and animals as well as to accelerate their applications as pharmaceuticals. The present study will provide a basis for a large preparation of ustilaginoidins from the rice FSBs, and also demonstrates that HSCCC is an efficient technique for preparative separation of bioactive compounds from natural materials.

## Supplementary Materials

Supplementary materials can be accessed at: http://www.mdpi.com/2072-6651/8/1/20/s1.
