# Supplementary material for: Preparative Separation of Main Ustilaginoidins from Rice False Smut Balls by High-Speed Counter-Current Chromatography
Source: Toxins (Basel). 2016 Jan 12;8(1):20. doi: 10.3390/toxins8010020 (PMC4728542; doi:10.3390/toxins8010020)
Supplement: Supplementary file 1 [file toxins-08-00020-s001.pdf]

Supplementary Materials: Preparative Separation of Main Ustilaginoidins from Rice False Smut Balls by High-Speed Counter-Current Chromatography

Weibo Sun, Xuejiao Dong, Dan Xu, Jiajia Meng, Xiaoxiang Fu, Xiaohan Wang, Daowan Lai, Ligang Zhou and Yang Liu

Table S1. The ^{1}H-nuclear magnetic resonance (^{1}H-NMR, 600 MHz) data of compounds **1–7**.

Position	1	2	3	4	5	6	7
2	-	4.42 ddq (11.9, 2.4, 5.9)	-	4.44 s	4.34 d (9.5)	-	4.33 d (11.7)
3	6.11 s	2.80 dd (17.4, 11.9)<br>2.72 dd (17.2, 2.4)	6.16 s	2.80 dd (17.2, 11.9)<br>2.68 d (15.6)	2.92 dd (17.3, 12.5)<br>2.61 d (17.2)	6.17 s	2.90 dd (17.4, 12.3)<br>2.59 dd (17.2, 2.4)
3′	6.11 s	5.90 s	6.16 s	5.64 s	5.70 s	6.17 s	5.68 s
7	6.62 s	6.50 s	6.59 s	6.46 s	6.47 s	6.61 s	6.45 s
7′	6.62 s	6.60 s	6.60 s	6.59 s	6.57 s	6.61 s	6.57 s
10	6.36 s	6.11 s	6.16 s	6.19 s	6.17 s	6.20 s	6.19 s
10′	6.36 s	6.38 s	6.20 s	6.21 s	6.20 s	6.20 s	6.19 s
2-CH_3	2.30 s	1.34 d (5.9)	2.24 s	1.27 d (6.2)	-	-	-
2′-CH_3	2.30 s	2.31 s	-	-	2.27 s	-	-
2-C$\underline{H_2}$OH	-	-	-	-	3.53 dd (18.4, 2.8)	4.27 d (4.4)	3.52 dd (18.7, 3.3)
2′-C$\underline{H_2}$OH	-	-	4.27 s	4.30 s	-	4.27 d (4.4)	4.29 d (2.5)
2-CH_2O$\underline{H}$	-	-	-	-	4.98 br. s	nd.	nd.
2′-CH_2O$\underline{H}$	-	-	5.76 s	5.79 s	-	nd.	nd.
6-OH	-	-	10.03 s	9.98 s	9.97 s	9.97 s	9.93 s
6′-OH	-	-	10.03 s	9.98 s	9.97 s	9.97 s	9.93 s
8-OH	-	-	9.82 s	9.82 s	9.78 s	9.82 s	9.78 s
8′-OH	-	-	9.82 s	9.82 s	9.79 s	9.82 s	9.78 s

Note: Compounds **1** and **2** were measured in acetone-d_6, and **3–7** were measured in DMSO-d_6. Chemical shifts were given on the δ (ppm) scale with TMS as the internal standard and coupling constants (J) were given in Hz. The letters s, d, dd, ddq and br.s mean singlet, doublet, doublet of doublets, doublet of doublet of quartets, and broad-singlet, respectively, in NMR spectrum. nd.: not detected.

Table S2. The ^{13}C-nuclear magnetic resonance (^{13}C-NMR, 150 MHz) data of compounds **1**–**7**.

Position	1	2	3	4	5	6	7
2	171.0	74.1	169.7	72.8	77.3	172.4	77.3
2′	171.0	171.0	172.4	172.4	169.8	172.4	172.4
3	106.3	43.6	103.4	42.8	37.7	103.3	37.7
3′	106.3	105.3	105.9	103.4	105.8	103.3	103.3
4	184.7	184.7	183.3	183.5	183.3	183.4	183.5
4′	184.7	199.4	183.4	198.1	198.0	183.4	198.0
4a	102.8	102.7	101.7	101.7	101.7	102.1	101.8
4a′	102.8	102.7	102.2	102.2	101.8	102.1	102.1
5	160.7	161.7	162.7	162.6	162.6	162.6	162.5
5′	160.7	163.2	162.7	164.9	164.7	162.6	164.6
5a	106.6	102.8	105.9	104.4	104.3	105.9	104.3
5a′	106.6	105.3	106.4	105.9	105.8	105.9	105.8
6	154.6	156.6	158.1	157.9	157.9	158.0	157.9
6′	154.6	159.6	158.1	158.9	158.8	158.0	158.8
7	99.6	99.6	98.0	98.2	98.0	98.2	98.2
7′	99.6	100.1	98.2	98.4	98.5	98.2	98.5
8	159.7	160.6	159.1	159.1	159.0	159.2	159.1
8′	159.7	161.6	159.2	160.1	160.1	159.2	160.0
9	107.0	106.6	106.4	106.6	106.6	106.4	106.6
9′	107.0	106.9	106.4	106.9	106.9	106.4	106.9
9a	141.2	141.1	139.8	139.9	139.7	139.9	139.8
9a′	141.2	143.1	140.0	141.6	141.6	139.9	141.6
10	101.5	100.7	100.8	100.0	100.0	100.9	100.0
10′	101.5	101.4	100.8	100.8	100.7	100.9	100.8
10a	153.4	153.4	151.7	151.6	151.9	151.6	151.6
10a′	153.4	154.6	152.0	155.0	154.8	151.6	154.8
2-CH_3	20.6	20.6	20.2	20.4	-	-	-
2′-CH_3	20.6	20.9	-	-	20.2	-	-
2-CH_2OH	-	-	-	-	62.7	59.8	59.8
2′-CH_2OH	-	-	59.8	59.8	-	59.8	62.7

Note: Compounds **1** and **2** were measured in acetone-d_6 and **3**–**7** were measured in DMSO-d_6. Chemical shifts were given on the *δ* (ppm) scale with TMS as the internal standard. a and a′ refer to the positions in the chemical structures shown in Figure 2.
